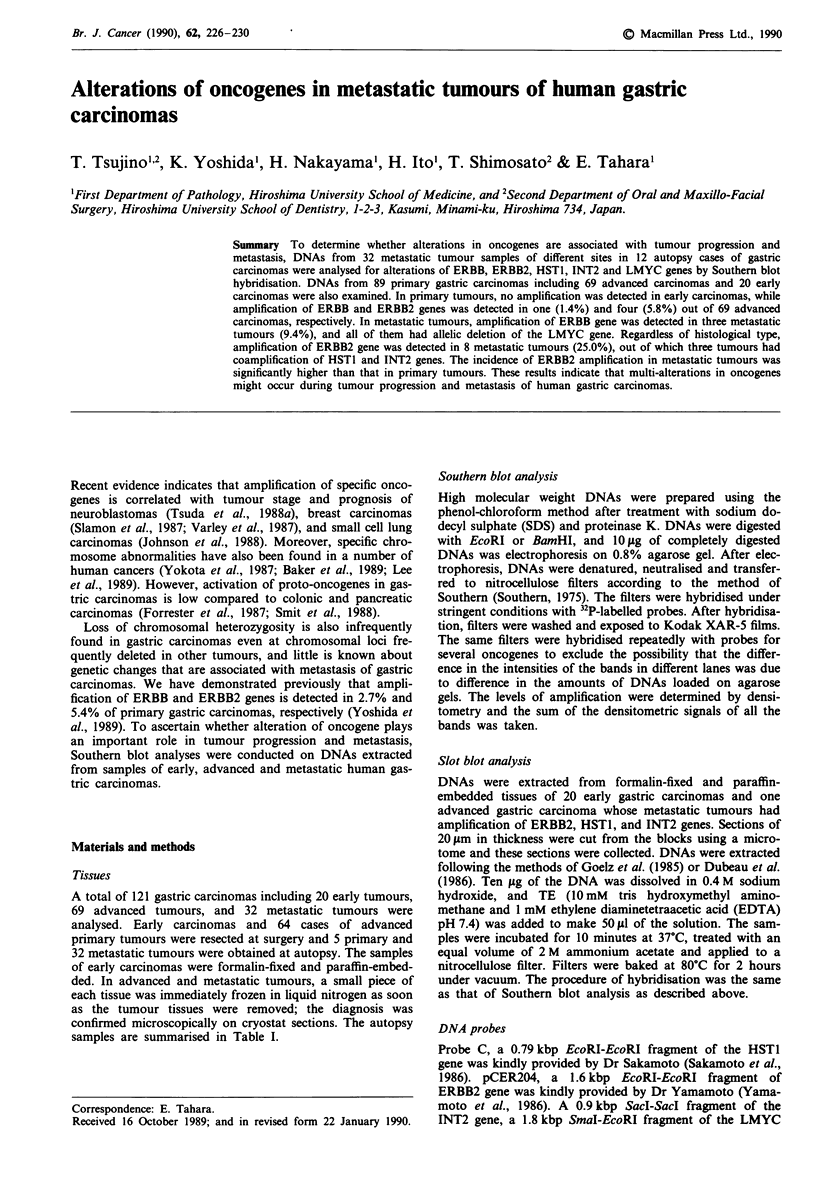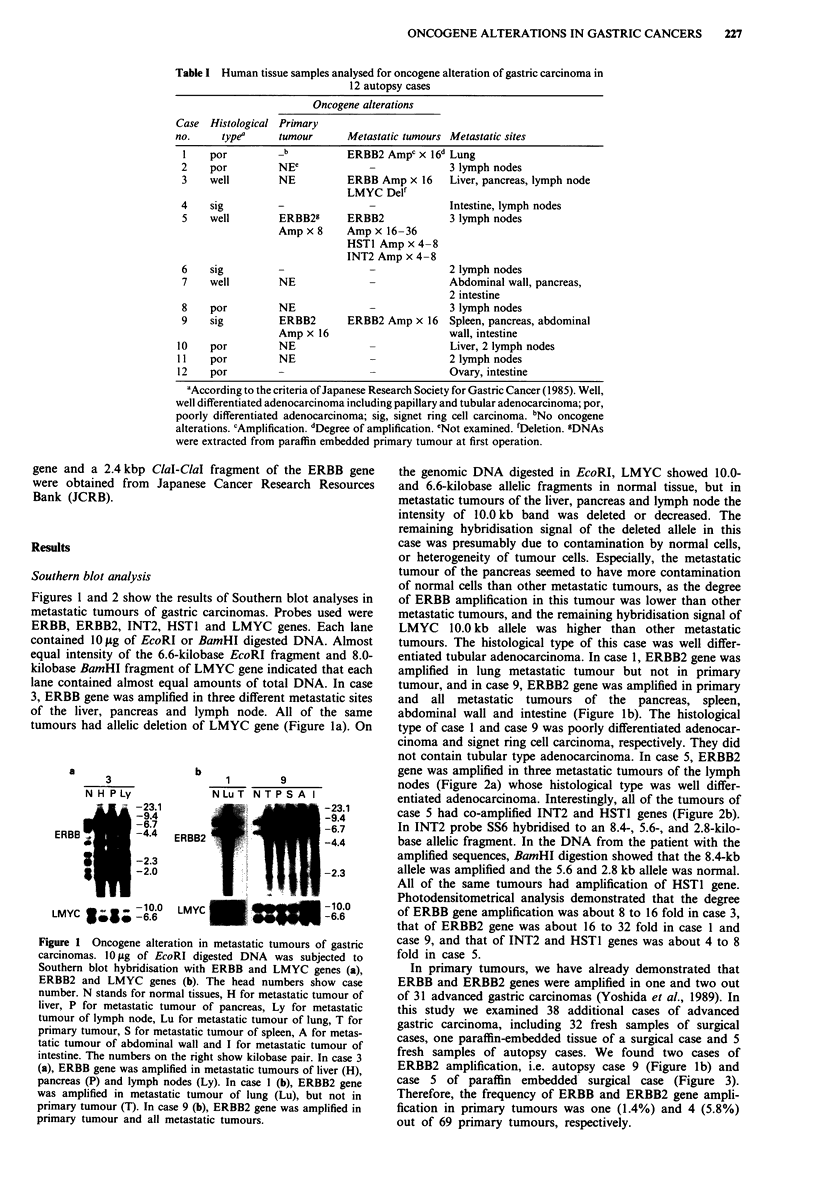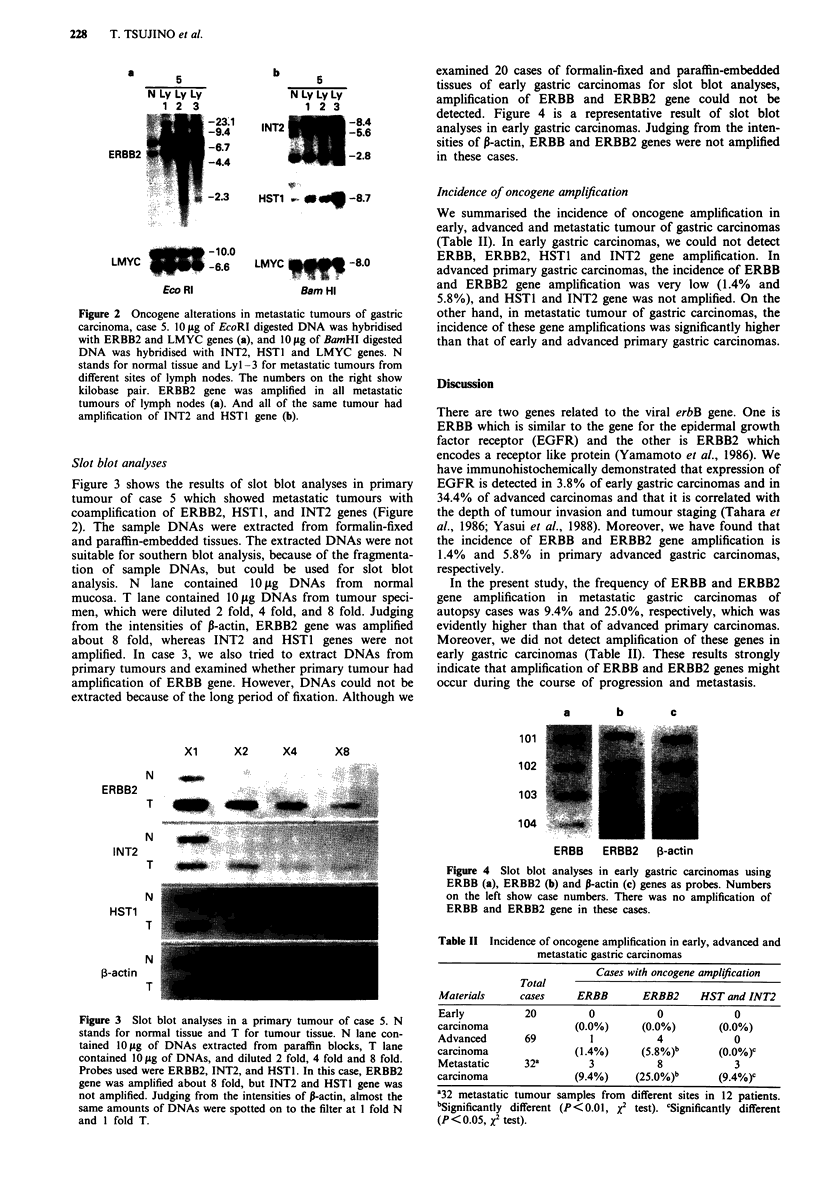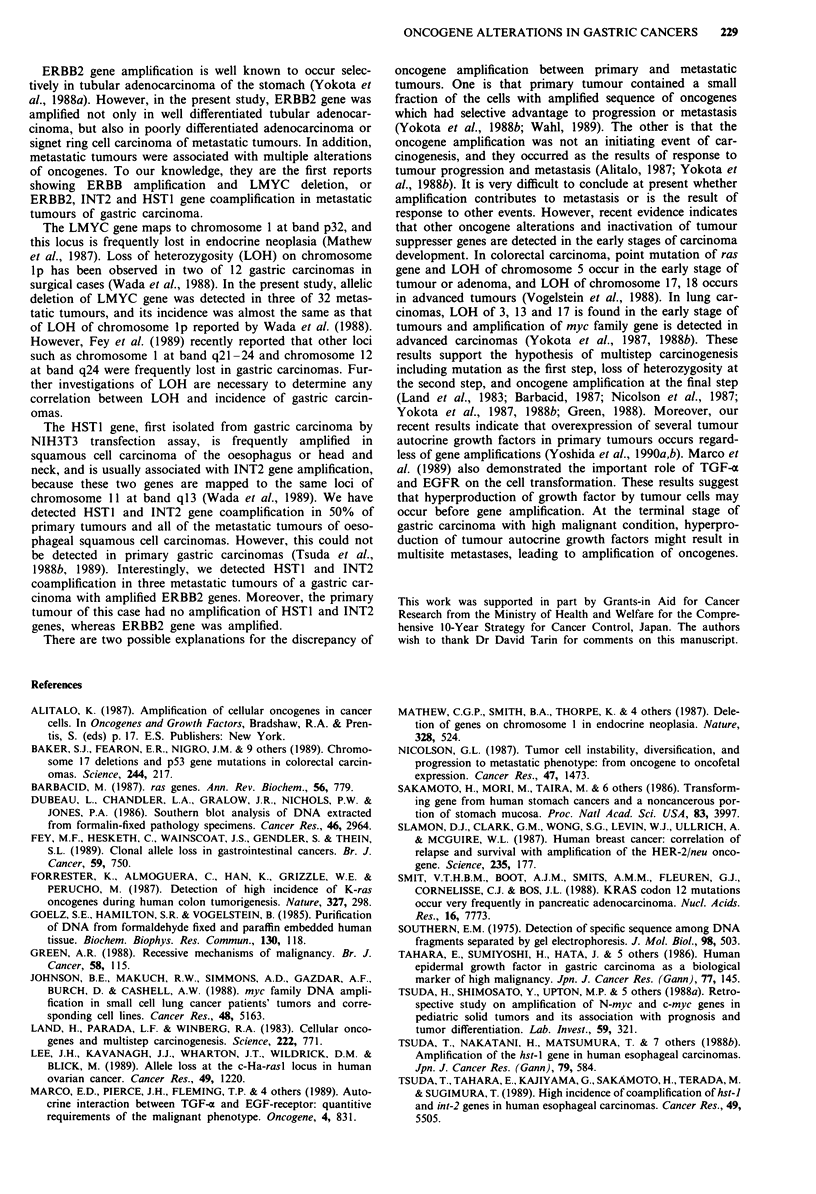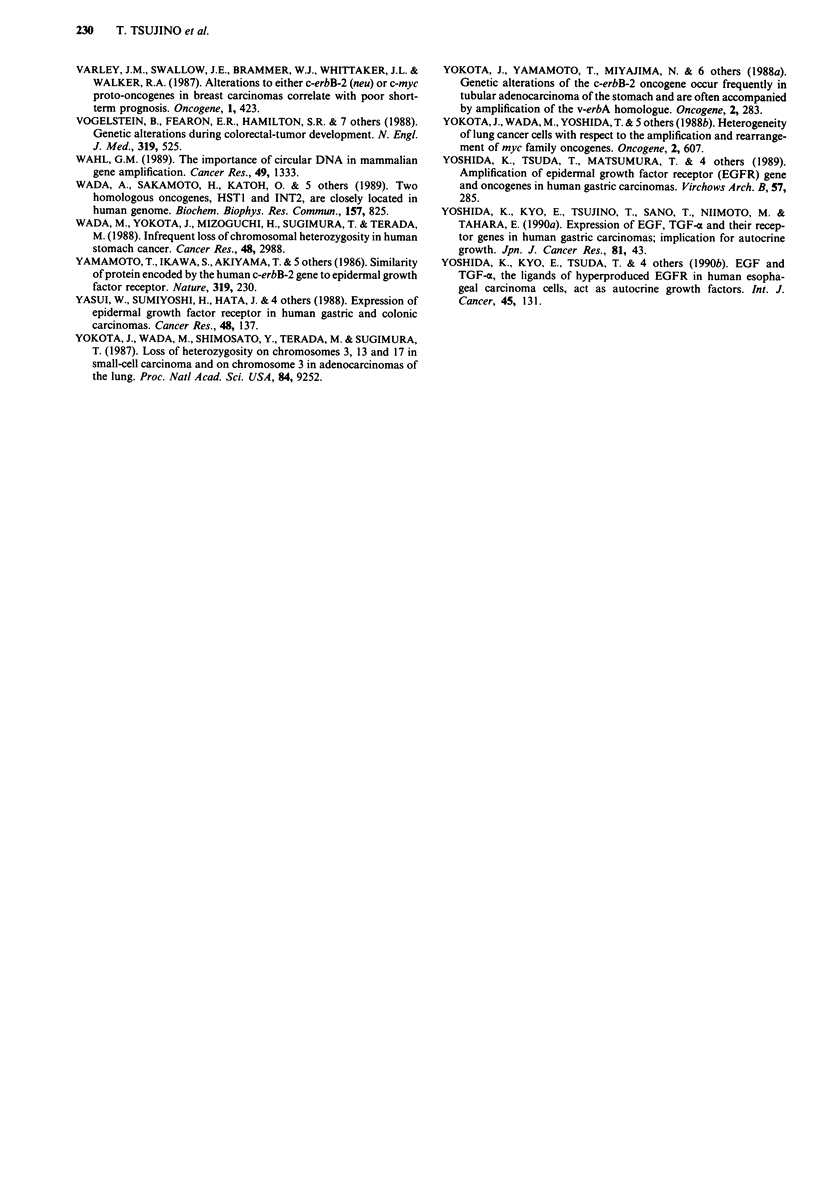# Alterations of oncogenes in metastatic tumours of human gastric carcinomas.

**DOI:** 10.1038/bjc.1990.265

**Published:** 1990-08

**Authors:** T. Tsujino, K. Yoshida, H. Nakayama, H. Ito, T. Shimosato, E. Tahara

**Affiliations:** First Department of Pathology, Hiroshima University School of Medicine, Japan.

## Abstract

**Images:**


*Br. J. Cancer* (1990), **62**, 226–230 © Macmillan Press Ltd., 1990

### Alterations of oncogenes in metastatic tumours of human gastric carcinomas

T. Tsujino[1,2], K. Yoshida[1], H. Nakayama[1], H. Ito[1], T. Shimosato[2] & E. Tahara[1]

[1]*First Department of Pathology, Hiroshima University School of Medicine, and* [2]*Second Department of Oral and Maxillo-Facial Surgery, Hiroshima University School of Dentistry, 1-2-3, Kasumi, Minami-ku, Hiroshima 734, Japan.*

**Summary** To determine whether alterations in oncogenes are associated with tumour progression and metastasis, DNAs from 32 metastatic tumour samples of different sites in 12 autopsy cases of gastric carcinomas were analysed for alterations of ERBB, ERBB2, HST1, INT2 and LMYC genes by Southern blot hybridisation. DNAs from 89 primary gastric carcinomas including 69 advanced carcinomas and 20 early carcinomas were also examined. In primary tumours, no amplification was detected in early carcinomas, while amplification of ERBB and ERBB2 genes was detected in one (1.4%) and four (5.8%) out of 69 advanced carcinomas, respectively. In metastatic tumours, amplification of ERBB gene was detected in three metastatic tumours (9.4%), and all of them had allelic deletion of the LMYC gene. Regardless of histological type, amplification of ERBB2 gene was detected in 8 metastatic tumours (25.0%), out of which three tumours had coamplification of HST1 and INT2 genes. The incidence of ERBB2 amplification in metastatic tumours was significantly higher than that in primary tumours. These results indicate that multi-alterations in oncogenes might occur during tumour progression and metastasis of human gastric carcinomas.

Recent evidence indicates that amplification of specific oncogenes is correlated with tumour stage and prognosis of neuroblastomas (Tsuda *et al.*, 1988*a*), breast carcinomas (Slamon *et al.*, 1987; Varley *et al.*, 1987), and small cell lung carcinomas (Johnson *et al.*, 1988). Moreover, specific chromosome abnormalities have also been found in a number of human cancers (Yokota *et al.*, 1987; Baker *et al.*, 1989; Lee *et al.*, 1989). However, activation of proto-oncogenes in gastric carcinomas is low compared to colonic and pancreatic carcinomas (Forrester *et al.*, 1987; Smit *et al.*, 1988).

Loss of chromosomal heterozygosity is also infrequently found in gastric carcinomas even at chromosomal loci frequently deleted in other tumours, and little is known about genetic changes that are associated with metastasis of gastric carcinomas. We have demonstrated previously that amplification of ERBB and ERBB2 genes is detected in 2.7% and 5.4% of primary gastric carcinomas, respectively (Yoshida *et al.*, 1989). To ascertain whether alteration of oncogene plays an important role in tumour progression and metastasis, Southern blot analyses were conducted on DNAs extracted from samples of early, advanced and metastatic human gastric carcinomas.

### Materials and methods

### *Tissues*

A total of 121 gastric carcinomas including 20 early tumours, 69 advanced tumours, and 32 metastatic tumours were analysed. Early carcinomas and 64 cases of advanced primary tumours were resected at surgery and 5 primary and 32 metastatic tumours were obtained at autopsy. The samples of early carcinomas were formalin-fixed and paraffin-embedded. In advanced and metastatic tumours, a small piece of each tissue was immediately frozen in liquid nitrogen as soon as the tumour tissues were removed; the diagnosis was confirmed microscopically on cryostat sections. The autopsy samples are summarised in Table I.

Correspondence: E. Tahara.
Received 16 October 1989; and in revised form 22 January 1990.

### *Southern blot analysis*

High molecular weight DNAs were prepared using the phenol-chloroform method after treatment with sodium dodecyl sulphate (SDS) and proteinase K. DNAs were digested with *Eco*RI or *Bam*HI, and 10 µg of completely digested DNAs was electrophoresis on 0.8% agarose gel. After electrophoresis, DNAs were denatured, neutralised and transferred to nitrocellulose filters according to the method of Southern (Southern, 1975). The filters were hybridised under stringent conditions with $^{32}$P-labelled probes. After hybridisation, filters were washed and exposed to Kodak XAR-5 films. The same filters were hybridised repeatedly with probes for several oncogenes to exclude the possibility that the difference in the intensities of the bands in different lanes was due to difference in the amounts of DNAs loaded on agarose gels. The levels of amplification were determined by densitometry and the sum of the densitometric signals of all the bands was taken.

### *Slot blot analysis*

DNAs were extracted from formalin-fixed and paraffin-embedded tissues of 20 early gastric carcinomas and one advanced gastric carcinoma whose metastatic tumours had amplification of ERBB2, HST1, and INT2 genes. Sections of 20 µm in thickness were cut from the blocks using a microtome and these sections were collected. DNAs were extracted following the methods of Goelz *et al.* (1985) or Dubeau *et al.* (1986). Ten µg of the DNA was dissolved in 0.4 M sodium hydroxide, and TE (10 mM tris hydroxymethyl aminomethane and 1 mM ethylene diaminetetraacetic acid (EDTA) pH 7.4) was added to make 50 µl of the solution. The samples were incubated for 10 minutes at 37°C, treated with an equal volume of 2 M ammonium acetate and applied to a nitrocellulose filter. Filters were baked at 80°C for 2 hours under vacuum. The procedure of hybridisation was the same as that of Southern blot analysis as described above.

### *DNA probes*

Probe C, a 0.79 kbp *Eco*RI-*Eco*RI fragment of the HST1 gene was kindly provided by Dr Sakamoto (Sakamoto *et al.*, 1986). pCER204, a 1.6 kbp *Eco*RI-*Eco*RI fragment of ERBB2 gene was kindly provided by Dr Yamamoto (Yamamoto *et al.*, 1986). A 0.9 kbp *Sac*I-*Sac*I fragment of the INT2 gene, a 1.8 kbp *Sma*I-*Eco*RI fragment of the LMYC

**Table I** Human tissue samples analysed for oncogene alteration of gastric carcinoma in 12 autopsy cases

| | | *Oncogene alterations* | | |
|---|---|---|---|---|
| *Case no.* | *Histological type*[a] | *Primary tumour* | *Metastatic tumours* | *Metastatic sites* |
| 1 | por | –[b] | ERBB2 Amp[c] × 16[d] | Lung |
| 2 | por | NE[e] | – | 3 lymph nodes |
| 3 | well | NE | ERBB Amp × 16<br>LMYC Del[f] | Liver, pancreas, lymph node |
| 4 | sig | – | – | Intestine, lymph nodes |
| 5 | well | ERBB2[g]<br>Amp × 8 | ERBB2<br>Amp × 16–36<br>HST1 Amp × 4–8<br>INT2 Amp × 4–8 | 3 lymph nodes |
| 6 | sig | – | – | 2 lymph nodes |
| 7 | well | NE | – | Abdominal wall, pancreas, 2 intestine |
| 8 | por | NE | – | 3 lymph nodes |
| 9 | sig | ERBB2<br>Amp × 16 | ERBB2 Amp × 16 | Spleen, pancreas, abdominal wall, intestine |
| 10 | por | NE | – | Liver, 2 lymph nodes |
| 11 | por | NE | – | 2 lymph nodes |
| 12 | por | – | – | Ovary, intestine |

[a]According to the criteria of Japanese Research Society for Gastric Cancer (1985). Well, well differentiated adenocarcinoma including papillary and tubular adenocarcinoma; por, poorly differentiated adenocarcinoma; sig, signet ring cell carcinoma. [b]No oncogene alterations. [c]Amplification. [d]Degree of amplification. [e]Not examined. [f]Deletion. [g]DNAs were extracted from paraffin embedded primary tumour at first operation.

gene and a 2.4 kbp *Cla*I-*Cla*I fragment of the ERBB gene were obtained from Japanese Cancer Research Resources Bank (JCRB).

### Results

### *Southern blot analysis*

Figures 1 and 2 show the results of Southern blot analyses in metastatic tumours of gastric carcinomas. Probes used were ERBB, ERBB2, INT2, HST1 and LMYC genes. Each lane contained 10 μg of *Eco*RI or *Bam*HI digested DNA. Almost equal intensity of the 6.6-kilobase *Eco*RI fragment and 8.0-kilobase *Bam*HI fragment of LMYC gene indicated that each lane contained almost equal amounts of total DNA. In case 3, ERBB gene was amplified in three different metastatic sites of the liver, pancreas and lymph node. All of the same tumours had allelic deletion of LMYC gene (Figure 1a). On

**Figure 1** Oncogene alteration in metastatic tumours of gastric carcinomas. 10 μg of *Eco*RI digested DNA was subjected to Southern blot hybridisation with ERBB and LMYC genes (**a**), ERBB2 and LMYC genes (**b**). The head numbers show case number. N stands for normal tissues, H for metastatic tumour of liver, P for metastatic tumour of pancreas, Ly for metastatic tumour of lymph node, Lu for metastatic tumour of lung, T for primary tumour, S for metastatic tumour of spleen, A for metastatic tumour of abdominal wall and I for metastatic tumour of intestine. The numbers on the right show kilobase pair. In case 3 (**a**), ERBB gene was amplified in metastatic tumours of liver (H), pancreas (P) and lymph nodes (Ly). In case 1 (**b**), ERBB2 gene was amplified in metastatic tumour of lung (Lu), but not in primary tumour (T). In case 9 (**b**), ERBB2 gene was amplified in primary tumour and all metastatic tumours.

the genomic DNA digested in *Eco*RI, LMYC showed 10.0- and 6.6-kilobase allelic fragments in normal tissue, but in metastatic tumours of the liver, pancreas and lymph node the intensity of 10.0 kb band was deleted or decreased. The remaining hybridisation signal of the deleted allele in this case was presumably due to contamination by normal cells, or heterogeneity of tumour cells. Especially, the metastatic tumour of the pancreas seemed to have more contamination of normal cells than other metastatic tumours, as the degree of ERBB amplification in this tumour was lower than other metastatic tumours, and the remaining hybridisation signal of LMYC 10.0 kb allele was higher than other metastatic tumours. The histological type of this case was well differentiated tubular adenocarcinoma. In case 1, ERBB2 gene was amplified in lung metastatic tumour but not in primary tumour, and in case 9, ERBB2 gene was amplified in primary and all metastatic tumours of the pancreas, spleen, abdominal wall and intestine (Figure 1b). The histological type of case 1 and case 9 was poorly differentiated adenocarcinoma and signet ring cell carcinoma, respectively. They did not contain tubular type adenocarcinoma. In case 5, ERBB2 gene was amplified in three metastatic tumours of the lymph nodes (Figure 2a) whose histological type was well differentiated adenocarcinoma. Interestingly, all of the tumours of case 5 had co-amplified INT2 and HST1 genes (Figure 2b). In INT2 probe SS6 hybridised to an 8.4-, 5.6-, and 2.8-kilobase allelic fragment. In the DNA from the patient with the amplified sequences, *Bam*HI digestion showed that the 8.4-kb allele was amplified and the 5.6 and 2.8 kb allele was normal. All of the same tumours had amplification of HST1 gene. Photodensitometrical analysis demonstrated that the degree of ERBB gene amplification was about 8 to 16 fold in case 3, that of ERBB2 gene was about 16 to 32 fold in case 1 and case 9, and that of INT2 and HST1 genes was about 4 to 8 fold in case 5.

In primary tumours, we have already demonstrated that ERBB and ERBB2 genes were amplified in one and two out of 31 advanced gastric carcinomas (Yoshida *et al.*, 1989). In this study we examined 38 additional cases of advanced gastric carcinoma, including 32 fresh samples of surgical cases, one paraffin-embedded tissue of a surgical case and 5 fresh samples of autopsy cases. We found two cases of ERBB2 amplification, i.e. autopsy case 9 (Figure 1b) and case 5 of paraffin embedded surgical case (Figure 3). Therefore, the frequency of ERBB and ERBB2 gene amplification in primary tumours was one (1.4%) and 4 (5.8%) out of 69 primary tumours, respectively.

**Figure 2** Oncogene alterations in metastatic tumours of gastric carcinoma, case 5. 10 μg of *Eco*RI digested DNA was hybridised with ERBB2 and LMYC genes (**a**), and 10 μg of *Bam*HI digested DNA was hybridised with INT2, HST1 and LMYC genes. N stands for normal tissue and Ly1–3 for metastatic tumours from different sites of lymph nodes. The numbers on the right show kilobase pair. ERBB2 gene was amplified in all metastatic tumours of lymph nodes (**a**). And all of the same tumour had amplification of INT2 and HST1 gene (**b**).

### *Slot blot analyses*

Figure 3 shows the results of slot blot analyses in primary tumour of case 5 which showed metastatic tumours with coamplification of ERBB2, HST1, and INT2 genes (Figure 2). The sample DNAs were extracted from formalin-fixed and paraffin-embedded tissues. The extracted DNAs were not suitable for southern blot analysis, because of the fragmentation of sample DNAs, but could be used for slot blot analysis. N lane contained 10 μg DNAs from normal mucosa. T lane contained 10 μg DNAs from tumour specimen, which were diluted 2 fold, 4 fold, and 8 fold. Judging from the intensities of β-actin, ERBB2 gene was amplified about 8 fold, whereas INT2 and HST1 genes were not amplified. In case 3, we also tried to extract DNAs from primary tumours and examined whether primary tumour had amplification of ERBB gene. However, DNAs could not be extracted because of the long period of fixation. Although we examined 20 cases of formalin-fixed and paraffin-embedded tissues of early gastric carcinomas for slot blot analyses, amplification of ERBB and ERBB2 gene could not be detected. Figure 4 is a representative result of slot blot analyses in early gastric carcinomas. Judging from the intensities of β-actin, ERBB and ERBB2 genes were not amplified in these cases.

**Figure 3** Slot blot analyses in a primary tumour of case 5. N stands for normal tissue and T for tumour tissue. N lane contained 10 μg of DNAs extracted from paraffin blocks, T lane contained 10 μg of DNAs, and diluted 2 fold, 4 fold and 8 fold. Probes used were ERBB2, INT2, and HST1. In this case, ERBB2 gene was amplified about 8 fold, but INT2 and HST1 gene was not amplified. Judging from the intensities of β-actin, almost the same amounts of DNAs were spotted on to the filter at 1 fold N and 1 fold T.

### *Incidence of oncogene amplification*

We summarised the incidence of oncogene amplification in early, advanced and metastatic tumour of gastric carcinomas (Table II). In early gastric carcinomas, we could not detect ERBB, ERBB2, HST1 and INT2 gene amplification. In advanced primary gastric carcinomas, the incidence of ERBB and ERBB2 gene amplification was very low (1.4% and 5.8%), and HST1 and INT2 gene was not amplified. On the other hand, in metastatic tumour of gastric carcinomas, the incidence of these gene amplifications was significantly higher than that of early and advanced primary gastric carcinomas.

### Discussion

There are two genes related to the viral *erb*B gene. One is ERBB which is similar to the gene for the epidermal growth factor receptor (EGFR) and the other is ERBB2 which encodes a receptor like protein (Yamamoto *et al*., 1986). We have immunohistochemically demonstrated that expression of EGFR is detected in 3.8% of early gastric carcinomas and in 34.4% of advanced carcinomas and that it is correlated with the depth of tumour invasion and tumour staging (Tahara *et al*., 1986; Yasui *et al*., 1988). Moreover, we have found that the incidence of ERBB and ERBB2 gene amplification is 1.4% and 5.8% in primary advanced gastric carcinomas, respectively.

In the present study, the frequency of ERBB and ERBB2 gene amplification in metastatic gastric carcinomas of autopsy cases was 9.4% and 25.0%, respectively, which was evidently higher than that of advanced primary carcinomas. Moreover, we did not detect amplification of these genes in early gastric carcinomas (Table II). These results strongly indicate that amplification of ERBB and ERBB2 genes might occur during the course of progression and metastasis.

**Figure 4** Slot blot analyses in early gastric carcinomas using ERBB (**a**), ERBB2 (**b**) and β-actin (**c**) genes as probes. Numbers on the left show case numbers. There was no amplification of ERBB and ERBB2 gene in these cases.

**Table II** Incidence of oncogene amplification in early, advanced and metastatic gastric carcinomas

| *Materials* | *Total cases* | *Cases with oncogene amplification* | | |
|---|---|---|---|---|
| | | *ERBB* | *ERBB2* | *HST and INT2* |
| Early carcinoma | 20 | 0 (0.0%) | 0 (0.0%) | 0 (0.0%) |
| Advanced carcinoma | 69 | 1 (1.4%) | 4 (5.8%)[b] | 0 (0.0%)[c] |
| Metastatic carcinoma | 32[a] | 3 (9.4%) | 8 (25.0%)[b] | 3 (9.4%)[c] |

[a]32 metastatic tumour samples from different sites in 12 patients. [b]Significantly different ($P<0.01$, $\chi^2$ test). [c]Significantly different ($P<0.05$, $\chi^2$ test).

ERBB2 gene amplification is well known to occur selectively in tubular adenocarcinoma of the stomach (Yokota *et al.*, 1988*a*). However, in the present study, ERBB2 gene was amplified not only in well differentiated tubular adenocarcinoma, but also in poorly differentiated adenocarcinoma or signet ring cell carcinoma of metastatic tumours. In addition, metastatic tumours were associated with multiple alterations of oncogenes. To our knowledge, they are the first reports showing ERBB amplification and LMYC deletion, or ERBB2, INT2 and HST1 gene coamplification in metastatic tumours of gastric carcinoma.

The LMYC gene maps to chromosome 1 at band p32, and this locus is frequently lost in endocrine neoplasia (Mathew *et al.*, 1987). Loss of heterozygosity (LOH) on chromosome 1p has been observed in two of 12 gastric carcinomas in surgical cases (Wada *et al.*, 1988). In the present study, allelic deletion of LMYC gene was detected in three of 32 metastatic tumours, and its incidence was almost the same as that of LOH of chromosome 1p reported by Wada *et al.* (1988). However, Fey *et al.* (1989) recently reported that other loci such as chromosome 1 at band q21-24 and chromosome 12 at band q24 were frequently lost in gastric carcinomas. Further investigations of LOH are necessary to determine any correlation between LOH and incidence of gastric carcinomas.

The HST1 gene, first isolated from gastric carcinoma by NIH3T3 transfection assay, is frequently amplified in squamous cell carcinoma of the oesophagus or head and neck, and is usually associated with INT2 gene amplification, because these two genes are mapped to the same loci of chromosome 11 at band q13 (Wada *et al.*, 1989). We have detected HST1 and INT2 gene coamplification in 50% of primary tumours and all of the metastatic tumours of oesophageal squamous cell carcinomas. However, this could not be detected in primary gastric carcinomas (Tsuda *et al.*, 1988*b*, 1989). Interestingly, we detected HST1 and INT2 coamplification in three metastatic tumours of a gastric carcinoma with amplified ERBB2 genes. Moreover, the primary tumour of this case had no amplification of HST1 and INT2 genes, whereas ERBB2 gene was amplified.

There are two possible explanations for the discrepancy of oncogene amplification between primary and metastatic tumours. One is that primary tumour contained a small fraction of the cells with amplified sequence of oncogenes which had selective advantage to progression or metastasis (Yokota *et al.*, 1988*b*; Wahl, 1989). The other is that the oncogene amplification was not an initiating event of carcinogenesis, and they occurred as the results of response to tumour progression and metastasis (Alitalo, 1987; Yokota *et al.*, 1988*b*). It is very difficult to conclude at present whether amplification contributes to metastasis or is the result of response to other events. However, recent evidence indicates that other oncogene alterations and inactivation of tumour suppresser genes are detected in the early stages of carcinoma development. In colorectal carcinoma, point mutation of *ras* gene and LOH of chromosome 5 occur in the early stage of tumour or adenoma, and LOH of chromosome 17, 18 occurs in advanced tumours (Vogelstein *et al.*, 1988). In lung carcinomas, LOH of 3, 13 and 17 is found in the early stage of tumours and amplification of *myc* family gene is detected in advanced carcinomas (Yokota *et al.*, 1987, 1988*b*). These results support the hypothesis of multistep carcinogenesis including mutation as the first step, loss of heterozygosity at the second step, and oncogene amplification at the final step (Land *et al.*, 1983; Barbacid, 1987; Nicolson *et al.*, 1987; Yokota *et al.*, 1987, 1988*b*; Green, 1988). Moreover, our recent results indicate that overexpression of several tumour autocrine growth factors in primary tumours occurs regardless of gene amplifications (Yoshida *et al.*, 1990*a,b*). Marco *et al.* (1989) also demonstrated the important role of TGF-α and EGFR on the cell transformation. These results suggest that hyperproduction of growth factor by tumour cells may occur before gene amplification. At the terminal stage of gastric carcinoma with high malignant condition, hyperproduction of tumour autocrine growth factors might result in multisite metastases, leading to amplification of oncogenes.

This work was supported in part by Grants-in Aid for Cancer Research from the Ministry of Health and Welfare for the Comprehensive 10-Year Strategy for Cancer Control, Japan. The authors wish to thank Dr David Tarin for comments on this manuscript.

### References

ALITALO, K. (1987). Amplification of cellular oncogenes in cancer cells. In *Oncogenes and Growth Factors*, Bradshaw, R.A. & Prentis, S. (eds) p. 17. E.S. Publishers: New York.

BAKER, S.J., FEARON, E.R., NIGRO, J.M. & 9 others (1989). Chromosome 17 deletions and p53 gene mutations in colorectal carcinomas. *Science*, **244**, 217.

BARBACID, M. (1987). *ras* genes. *Ann. Rev. Biochem.*, **56**, 779.

DUBEAU, L., CHANDLER, L.A., GRALOW, J.R., NICHOLS, P.W. & JONES, P.A. (1986). Southern blot analysis of DNA extracted from formalin-fixed pathology specimens. *Cancer Res.*, **46**, 2964.

FEY, M.F., HESKETH, C., WAINSCOAT, J.S., GENDLER, S. & THEIN, S.L. (1989). Clonal allele loss in gastrointestinal cancers. *Br. J. Cancer*, **59**, 750.

FORRESTER, K., ALMOGUERA, C., HAN, K., GRIZZLE, W.E. & PERUCHO, M. (1987). Detection of high incidence of K-*ras* oncogenes during human colon tumorigenesis. *Nature*, **327**, 298.

GOELZ, S.E., HAMILTON, S.R. & VOGELSTEIN, B. (1985). Purification of DNA from formaldehyde fixed and paraffin embedded human tissue. *Biochem. Biophys. Res. Commun.*, **130**, 118.

GREEN, A.R. (1988). Recessive mechanisms of malignancy. *Br. J. Cancer*, **58**, 115.

JOHNSON, B.E., MAKUCH, R.W., SIMMONS, A.D., GAZDAR, A.F., BURCH, D. & CASHELL, A.W. (1988). *myc* family DNA amplification in small cell lung cancer patients' tumors and corresponding cell lines. *Cancer Res.*, **48**, 5163.

LAND, H., PARADA, L.F. & WINBERG, R.A. (1983). Cellular oncogenes and multistep carcinogenesis. *Science*, **222**, 771.

LEE, J.H., KAVANAGH, J.J., WHARTON, J.T., WILDRICK, D.M. & BLICK, M. (1989). Allele loss at the c-Ha-*ras*1 locus in human ovarian cancer. *Cancer Res.*, **49**, 1220.

MARCO, E.D., PIERCE, J.H., FLEMING, T.P. & 4 others (1989). Autocrine interaction between TGF-α and EGF-receptor: quantitive requirements of the malignant phenotype. *Oncogene*, **4**, 831.

MATHEW, C.G.P., SMITH, B.A., THORPE, K. & 4 others (1987). Deletion of genes on chromosome 1 in endocrine neoplasia. *Nature*, **328**, 524.

NICOLSON, G.L. (1987). Tumor cell instability, diversification, and progression to metastatic phenotype: from oncogene to oncofetal expression. *Cancer Res.*, **47**, 1473.

SAKAMOTO, H., MORI, M., TAIRA, M. & 6 others (1986). Transforming gene from human stomach cancers and a noncancerous portion of stomach mucosa. *Proc. Natl Acad. Sci. USA*, **83**, 3997.

SLAMON, D.J., CLARK, G.M., WONG, S.G., LEVIN, W.J., ULLRICH, A. & MCGUIRE, W.L. (1987). Human breast cancer: correlation of relapse and survival with amplification of the HER-2/*neu* oncogene. *Science*, **235**, 177.

SMIT, V.T.H.B.M., BOOT, A.J.M., SMITS, A.M.M., FLEUREN, G.J., CORNELISSE, C.J. & BOS, J.L. (1988). KRAS codon 12 mutations occur very frequently in pancreatic adenocarcinoma. *Nucl. Acids. Res.*, **16**, 7773.

SOUTHERN, E.M. (1975). Detection of specific sequence among DNA fragments separated by gel electrophoresis. *J. Mol. Biol.*, **98**, 503.

TAHARA, E., SUMIYOSHI, H., HATA, J. & 5 others (1986). Human epidermal growth factor in gastric carcinoma as a biological marker of high malignancy. *Jpn. J. Cancer Res. (Gann)*, **77**, 145.

TSUDA, H., SHIMOSATO, Y., UPTON, M.P. & 5 others (1988*a*). Retrospective study on amplification of N-*myc* and c-*myc* genes in pediatric solid tumors and its association with prognosis and tumor differentiation. *Lab. Invest.*, **59**, 321.

TSUDA, T., NAKATANI, H., MATSUMURA, T. & 7 others (1988*b*). Amplification of the *hst*-1 gene in human esophageal carcinomas. *Jpn. J. Cancer Res. (Gann)*, **79**, 584.

TSUDA, T., TAHARA, E., KAJIYAMA, G., SAKAMOTO, H., TERADA, M. & SUGIMURA, T. (1989). High incidence of coamplification of *hst-1* and *int-2* genes in human esophageal carcinomas. *Cancer Res.*, **49**, 5505.

VARLEY, J.M., SWALLOW, J.E., BRAMMER, W.J., WHITTAKER, J.L. & WALKER, R.A. (1987). Alterations to either c-*erb*B-2 (*neu*) or c-*myc* proto-oncogenes in breast carcinomas correlate with poor short-term prognosis. *Oncogene*, **1**, 423.

VOGELSTEIN, B., FEARON, E.R., HAMILTON, S.R. & 7 others (1988). Genetic alterations during colorectal-tumor development. *N. Engl. J. Med.*, **319**, 525.

WAHL, G.M. (1989). The importance of circular DNA in mammalian gene amplification. *Cancer Res.*, **49**, 1333.

WADA, A., SAKAMOTO, H., KATOH, O. & 5 others (1989). Two homologous oncogenes, HST1 and INT2, are closely located in human genome. *Biochem. Biophys. Res. Commun.*, **157**, 825.

WADA, M., YOKOTA, J., MIZOGUCHI, H., SUGIMURA, T. & TERADA, M. (1988). Infrequent loss of chromosomal heterozygosity in human stomach cancer. *Cancer Res.*, **48**, 2988.

YAMAMOTO, T., IKAWA, S., AKIYAMA, T. & 5 others (1986). Similarity of protein encoded by the human c-*erb*B-2 gene to epidermal growth factor receptor. *Nature*, **319**, 230.

YASUI, W., SUMIYOSHI, H., HATA, J. & 4 others (1988). Expression of epidermal growth factor receptor in human gastric and colonic carcinomas. *Cancer Res.*, **48**, 137.

YOKOTA, J., WADA, M., SHIMOSATO, Y., TERADA, M. & SUGIMURA, T. (1987). Loss of heterozygosity on chromosomes 3, 13 and 17 in small-cell carcinoma and on chromosome 3 in adenocarcinomas of the lung. *Proc. Natl Acad. Sci. USA*, **84**, 9252.

YOKOTA, J., YAMAMOTO, T., MIYAJIMA, N. & 6 others (1988*a*). Genetic alterations of the c-*erb*B-2 oncogene occur frequently in tubular adenocarcinoma of the stomach and are often accompanied by amplification of the v-*erb*A homologue. *Oncogene*, **2**, 283.

YOKOTA, J., WADA, M., YOSHIDA, T. & 5 others (1988*b*). Heterogeneity of lung cancer cells with respect to the amplification and rearrangement of *myc* family oncogenes. *Oncogene*, **2**, 607.

YOSHIDA, K., TSUDA, T., MATSUMURA, T. & 4 others (1989). Amplification of epidermal growth factor receptor (EGFR) gene and oncogenes in human gastric carcinomas. *Virchows Arch. B*, **57**, 285.

YOSHIDA, K., KYO, E., TSUJINO, T., SANO, T., NIIMOTO, M. & TAHARA, E. (1990*a*). Expression of EGF, TGF-α and their receptor genes in human gastric carcinomas; implication for autocrine growth. *Jpn. J. Cancer Res.*, **81**, 43.

YOSHIDA, K., KYO, E., TSUDA, T. & 4 others (1990*b*). EGF and TGF-α, the ligands of hyperproduced EGFR in human esophageal carcinoma cells, act as autocrine growth factors. *Int. J. Cancer*, **45**, 131.